# Risk Factors for Cerebrovascular Disease Mortality among the Elderly in Beijing: A Competing Risk Analysis

**DOI:** 10.1371/journal.pone.0087884

**Published:** 2014-02-04

**Authors:** Zhe Tang, Tao Zhou, Yanxia Luo, Changchun Xie, Da Huo, Lixin Tao, Lei Pan, Fei Sun, Huiping Zhu, Xinghua Yang, Wei Wang, Aoshuang Yan, Xia Li, Xiuhua Guo

**Affiliations:** 1 Beijing Geriatric Healthcare Center, Xuan Wu Hospital, Capital Medical University, Beijing, China; 2 School of Public Health, Capital Medical University, Beijing, China; 3 Beijing Municipal Key Laboratory of Clinical Epidemiology, Beijing, China; 4 Division of Epidemiology and Biostatistics, Department of Environmental Health, University of Cincinnati, Cincinnati, Ohio, United States of America; 5 Center for Clinical and Translational Science and Training, University of Cincinnati, Cincinnati, Ohio, United States of America; 6 Institute for Infectious Disease and Endemic Disease Control, Beijing Center for Disease Prevention and Control, Beijing, China; 7 School of Medical Science, Edith Cowan University, Perth, Australia; 8 Beijing Municipal Science and Technology Commission, Beijing, China; 9 Epidemiology and Public Health, University College Cork, Cork, Ireland; University of Leicester, United Kingdom

## Abstract

**Objective:**

To examine the associations of combined lifestyle factors and physical conditions with cerebrovascular diseases (CBVD) mortality, after accounting for competing risk events, including death from cardiovascular diseases, cancers and other diseases.

**Methods:**

Data on 2010 subjects aged over 55 years were finally analyzed using competing risk models. All the subjects were interviewed by the Beijing Longitudinal Study of Aging (BLSA), in China, between 1 January 1992 and 30 August 2009.

**Results:**

Elderly females were at a lower risk of death from CBVD than elderly males (HR = 0.639, 95% CI = 0.457–0.895). Increasing age (HR = 1.543, 95% CI = 1.013–2.349), poor self-rated health (HR = 1.652, 95% CI = 1.198–2.277), hypertension (HR = 2.201, 95% CI = 1.524–3.178) and overweight (HR = 1.473, 95% CI = 1.013–2.142) or obesity (HR = 1.711, 95% CI = 1.1754–2.490) was associated with higher CBVD mortality risk. Normal cognition function (HR = 0.650, 95% CI = 0.434–0.973) and living in urban (HR = 0.456, 95% CI = 0.286–0.727) was associated with lower CBVD mortality risk. Gray’s test also confirmed the cumulative incidence (CIF) of CBVD was lower in the ‘married’ group than those without spouse, and the mortality was lowest in the ‘nutrition sufficient’ group among the ‘frequent consumption of meat group’ and the ‘medial type group’ (*P* value<0.001).

**Conclusions:**

CBVD mortality was associated with gender, age, blood pressure, residence, BMI, cognitive function, nutrition and the result of self-rated health assessment in the elderly in Beijing, China.

## Introduction

The incidence of cerebrovascular diseases (CBVD) has increased by 100% in developing nations and it is the leading cause of sustained neurological disability in the world [1]. Furthermore, CBVD is the second leading cause of death in middle-income countries, accounting for 12.8% of all-cause mortality according to the World Health Organization (WHO) [2]. An epidemiological survey confirmed that the standardized death rate for CBVD has reached 120.1 *per* 100,000 in China [3]. With increasing age, the crude death rate of CBVD showed a fast growth [4].

The high mortality and disability rates from CBVD not only affected the health and quality of life of the victims, but also caused heavy economic and mental burdens for families and the society [5]. Meanwhile, the burden of CBVD is likely to increase substantially in the future because of the aging population and changes in lifestyle.

However, studies on how lifestyle and/or other factors integrally function in the homes of the elderly are rare, especially in Asia. This study was to develop a comprehensive model for CBVD mortality incorporating the effects of lifestyle factors and physical conditions, among the population of an18-year cohort study conducted by BLSA. We aimed to incorporate the competing events of the alternate outcomes into a competing-risk analysis [6]–[9].

## Materials and Methods

### Ethical Approval

All participants were asked to sign an informed consent form and the ethics committee w of Xuanwu Hospital Capital Medical University approved the project. Written informed consents were obtained for every subject.

### Study Population

The BLSA, a community-based cohort study begun in August 1992, is an on-going, prospective study in Beijing, China, hosted by Xuanwu Hospital. A three-stage stratification random clustering procedure was used to ensure the representativeness of Beijing elderly in general. The procedures for sampling and data collection were described in detail elsewhere [10] [11]. Firstly, Beijing consists of 18 administrative districts that were divided into three categories according to the degree of urbanization and economic status: main urban areas, suburbs and mountain areas. Three districts: Xuanwu (urban), Daxing (suburban) and Huairou (rural) were selected as representing the average age, education and economic level for the category. Secondly, specific neighborhoods (streets or villages) were randomly selected from these three districts. Thirdly, a predetermined number of subjects were selected from these neighborhood units and villages using a systematic sampling method. Finally, 3,257 subjects were selected. In this analysis 2,101 subjects whose serum biochemical indices were measured after informed consent were chosen. The enrolled and the omitted subjects were compared to assess enrolment bias, the differences in characteristics between these two groups were not statistically significant.

A follow-up questionnaire, including demographic characteristics, socio-economic status, and health related issues, was sent to participants every two or three years after enrollment. All participants were asked to sign an informed consent form and the ethics committee of Xuanwu Hospital Capital Medical University approved the project. Written informed consents were obtained for every subject. The questionnaires were completed in the respondents’ homes by trained interviewers, usually nurses, doctors or senior medical students. They also completed the questionnaire on behalf of illiterate participants.

### Assessment of Risk Factors

Besides the basic characteristics, life style and physical condition, some scales were also measured in more detail: activities of Daily Living (ADL) scale, Center for Epidemiological Studies Depression (CES-D) scale, and Mini-Mental State Examination scale (MMSE). Baseline values were used in this analysis to minimize the potential of clinical or subclinical diseases affecting the risk factor status.

Age was categorized in 10-year age categories from 55 through 75 years plus an age group containing persons older than 75. According to height, and weight, BMI (Body Mass Index) was computed and grouped into four categorized: thin, normal, overweight and obesity [12]. Blood samples were collected after an overnight fasting of at least 12 hours. Glucose, total cholesterol (TC), high density lipoprotein (HDL), low density lipoprotein (LDL), and triglycerides (TG) were subsequently measured. Subjects were divided into normal or abnormal groups according to the standard of diabetes mellitus [13] and dyslipidemia [14]. Blood pressure (BP) was measured on the right arm of subjects seated and at rest for at least 10 min by a trained nurse. BP was categorized into three groups: normal, critical and high blood pressure. The questionnaire also included the frequency of taking exercise: if the elderly exercised regularly (almost every day), then this was defined exercising frequently. The related activities included walking, Tai Chi, running/jogging, Qi Gong, dancing, *etc*.

Dietary habits included the categorization of the oil, the frequency and consumption of eggs, milk, fish, vegetables, coffee, tea, *etc*. Then the latent class model (LCA) was conducted and the best model was selected according to the value of Bayesian information criterion (BIC). Dietary habits were divided into three latent categories (groups). Based on *a posterior* probability (representing the frequency of food intake), the dietary structure was classified as: sufficient nutrients (Group 1), intermediate-type (Group 2) and meat-based diet (Group3). Generally, the intake of milk, fruits, bean products, and eggs were lower in Group 2 and 3. [15].

Physical limitation was assessed using the 12 items in ADL, Instrumental Activities of Daily Living (IADL) and Basic Activities of Daily living (BADL) surveys [16]. The subjects were categorized into complete ability or disability based on these two scales, respectively. The CESD was used to assess depression. The total score was 60 and the standard cut-off value was 16 [17]. A higher score corresponded to a more severe condition. The MMSE was used for differentiating cognitive levels [18]: this is closely related to the level of education; people of low intelligence or poor education may score poorly on this examination in the absence of cognitive impairment, and well-educated people may score well despite having cognitive impairment. The critical threshold values are: illiterate>17, primary school education>20, secondary or higher education>24.

### Outcome Assessment

The outcome was death from all causes, occurring after the return of the 1992 questionnaire but before 31 December 2009. Survival status was determined through interviews with surviving household members and with neighbors when surviving household members were unavailable. The information was verified by a subset of participants based on household registration records and their death records. CBVD mortality was defined as the primary cause of death as indicated by the International Classification of Disease (ICD), ninth revision ICD-9 or ICD-10. Death from cardiovascular diseases, cancers and other causes consisted of competing events.

### Statistical Analysis

A few values from biochemical serums were missing, thus a multiple imputation (MI) was performed to impute the missing information. According to the data distribution, the Markov Chain Monte Carlo (MCMC) method was chosen to avoid the loss of generality. The MI procedure in the SAS software package (Version 9.2; SAS Institute, Chicago, IL, USA) was used [19].

Time of follow-up accrued from the return date of the 2009 questionnaire until either death, loss of follow up or the end of follow up (31 December, 2009), whichever came first. We fitted a competing risk model to compute hazard ratios (HR) and 95% confidence intervals (95% CI) for the associations between each risk factor and CBVD mortality. When the multivariate model was conducted, we tried to perform a univariate analysis (*P* value<0.3) as the criteria for inclusion of risk factors in the final multivariate model. The competing risk model was performed in R software.

## Results

**Figure 1:**
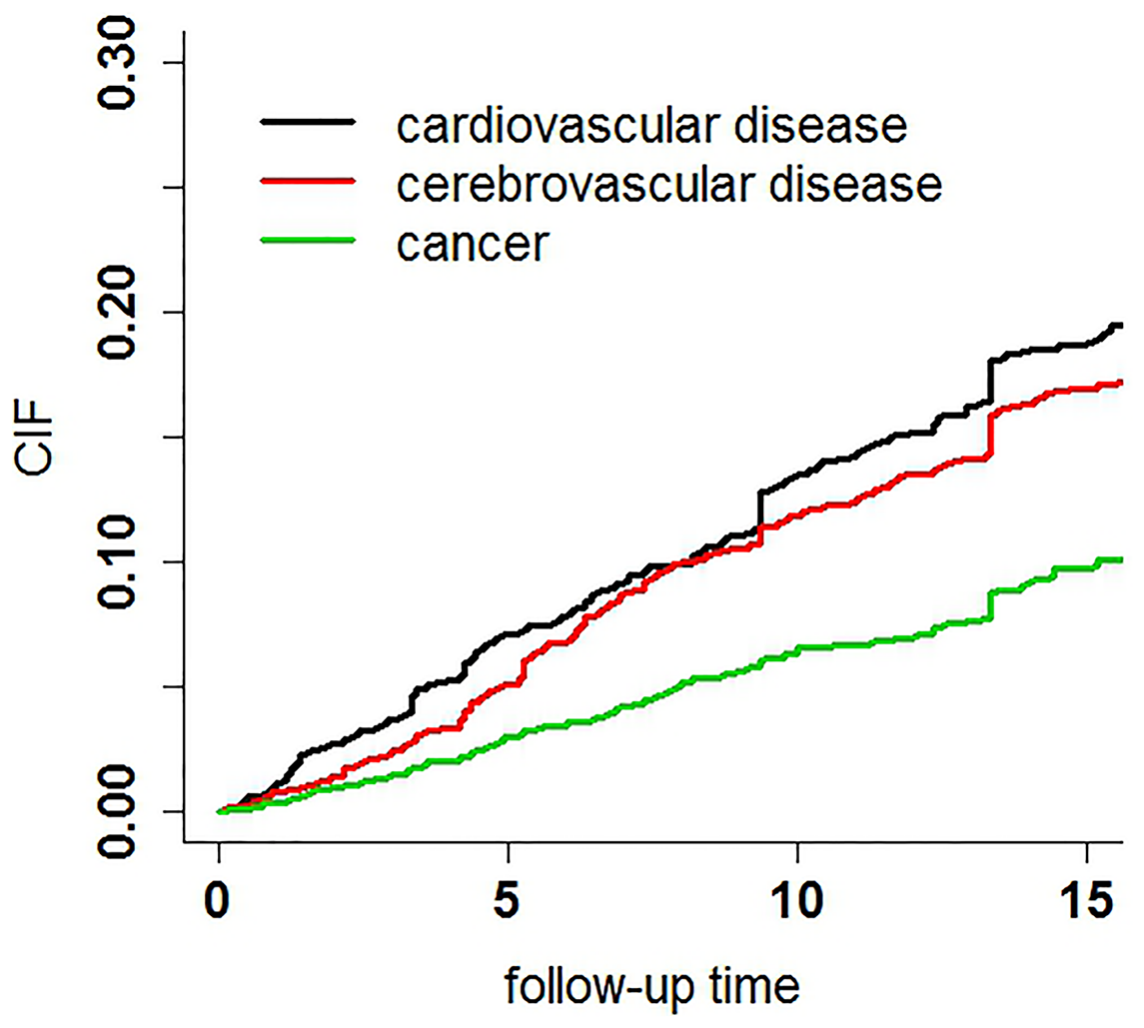
CIFs for three main outcomes: CVD, CBVD and cancer.

Among the 2,101 community dwellers aged 55 years or over in 1992, 91 subjects were excluded because of missing information regarding height, weight, some scales, *etc*. In the end, a total of 2,010 participants were included in the analysis. 374 subjects had missing data in some serum biochemical profiles and were made up by MCMC. By the end of the follow-up in 2009, there were 356 surviving subjects, 586 missing subjects, and 1068 deaths. Among the 1068 deaths, 273 were caused by cardiovascular diseases (25.5%), 246 by cerebrovascular diseases (23.0%), 140 by cancer (13.1%), and 409 were from other causes (38.4%) (Table 1). At the end of follow up, considering the competing risks, the CIF of CVD death was 0.19, for CBVD it was 0.17, and cancer was 0.10 (Figure 1).

**Table 1:** Characteristics of subjects in Beijing between 1992 and 2009.

Characteristic	Total subjects (%)	Total deaths (%)	Cerebrovasculardisease deaths (%)
Total	2010(100)	1068(100)	246 (100)
Sex			
male	987(49.104)	545 (51.030)	141 (57.317)
female	1023(50.896)	523 (48.970)	105 (42.683)
Age-group			
55–65	705 (35.075)	246 (23.034)	66 (26.829)
66–75	728 (36.219)	408 (38.202)	106(43.089)
≥76	577 (28.706)	414 (38.764)	74 (30.081)
Smoke			
no	1417(70.497)	738 (69.101)	162 (65.854)
yes	593 (29.503)	330 (30.899)	84 (34.146)
Drink			
no	1569(78.060)	832 (42.24)	182(73.984)
yes	441(21.940)	236 (22.097)	64 (26.016)
Depression			
no	1649(82.040)	849 (79.494)	191(77.642)
yes	361(17.960)	219 (20.506)	55 (22.358)
Sadevent			
no	1364(67.861)	724 (67.790)	158(64.228)
yes	646(32.139)	344 (32.210)	88 (35.772)
Exercise			
no	806(40.100)	419 (39.232)	95 (38.211)
yes	1204(59.901)	649 (60.768)	151(61.382)
Basic ADL			
normal	1936(74.378)	1010(94.570)	231(93.902)
disability	74 (25.622)	58 (5.431)	15 (6.098)
Instrumental ADL			
normal	1495(74.378)	696 (65.169)	161(65.447)
disability	515(25.622)	372 (34.831)	85 (34.553)
Maritalstatus			
have a spouse	1354(67.363)	645 (60.393)	163(66.260)
mateless	656(32.637)	423 (39.607)	83 (33.740)
MMSE			
abnormal	383(19.055)	228 (21.348)	58 (23.577)
normal	1627(80.945)	840 (78.652)	188(76.423)
Self-assessment of health			
health	1639(81.542)	826 (77.341)	176(71.545)
not health	371(18.458)	242 (22.659)	70 (8.130)
Diabetes			
no	1698(84.478)	876 (82.022)	205(83.333)
yes	312(15.522)	192 (17.978)	41 (16.667)
Bloodlipid			
abnormal	500 (24.876)	251 (23.502)	55 (22.358)
normal	1510(75.124)	817 (76.498)	191(77.642)
Blood pressure			
sbp≤120 or dbp≤80	551(27.413)	245 (22.940)	37 (15.041)
sbp>120 or dbp>80	255(12.687)	106 (9.925)	25 (10.163)
sbp≥140 or dbp≥90	1240(61.692)	717 (67.135)	184(74.797)
Education level			
college degree or above	137(6.816)	48 (4.494)	10 (4.065)
high school diploma	96(4.875)	34 (3.184)	6 (2.439)
junior diploma	165(8.209)	73 (6.835)	17 (6.911)
primary school	579(28.806)	283 (26.498)	73 (29.675)
illiterate	1033(51.393)	630 (58.989)	140(56.911)
Body mass index			
normal	1102(54.826)	579 (54.213)	131(53.252)
thin	376(18.706)	239 (22.378)	32 (13.008)
overweight	276(13.731)	118 (11.049)	40 (16.260)
obesity	256(12.736)	132 (12.360)	43 (17.480)
Area			
mountain	283(14.080)	168 (15.730)	54 (21.951)
rural	508(25.274)	338(31.648)	88(35.772)
urban	1219(60.647)	562 (52.622)	104(42.276)
Diet			
middle status	1016(50.547)	569 (53.277)	138(56.098)
nutritionally adequate	693(34.478)	300 (28.090)	54 (21.951)
extra serving of meat	301(14.975)	199 (18.633)	54 (21.951)

### Results from Univariate Analysis of the Total Population

Table 2 showed the association of each variable with CBVD mortality. After considering competing risk events, gender, BADL, IADL, cognitive function assessed by MMSE, self-assessment of health, age group, BP, BMI, area of primary residence, and dietary habits were associated with CBVD mortality. It was found that female individuals were at a lower risk than male individuals (HR = 0.690, 95% CI = 0.537–0.888). Those with normal cognitive function were at a lower risk (HR = 0.741, 95% CI = 0.552–0.995) than those with diminished cognitive function. The mortality of the disabled, as assessed by BADL or IADL, and poor self-health assessment were respectively higher than the subjects in normal BADL group (HR = 1.870, 95% CI = 1.091–3.194), normal IADL group (HR = 1.670, 95% CI = 1.285–2.173), and normal self-health assessment (HR = 1.840, 95% CI = 1.413–2.432).

**Table 2:** Predictors of mortality from CBVDs, using competing risks models.

	Univariate analysis			Multivariate analysis				
Variables	*P* value	HR	95% CI	*P* value	HR	95% CI		
Sex(male)	0.004	0.690	0.537–0.888	<0.001	0.639	0.457–0.895		
Weight	0.511	1.010	0.993–1.010	–	–	–		
Depression(normal)	0.060	1.330	0.988–1.811	0.639	1.085	0.772–1.523		
Smoke(no smoking)	0.150	0.826	0.637–1.071	0.201	0.829	0.623–1.103		
Drink(no drinking)	0.110	0.795	0.599–1.061	0.853	1.031	0.744–1.429		
Sad–event(experienced)	0.290	0.871	0.672–1.130	0.214	0.842	0.645–1.099		
Excise(experience)	0.410	0.899	0.696–1.160	–	–			
BADL(abled)	0.022	1.870	1.091–3.194	0.468	1.245	0.684–2.266		
IADL(abled)	<0.001	1.670	1.285–2.173	0.579	1.104	0.780–1.564		
Maritalstatus(mateless)	0.350	1.130	0.871–1.476	–	–			
MMSE(abnormal)	0.046	0.741	0.552–0.995	0.036	0.650	0.434–0.973		
Self-assessment of health(health)	<0.001	1.840	1.413–2.432	0.002	1.652	1.198–2.277		
Blood-lipid(normal)	0.334	0.863	0.639–1.161	–	–	–		
Diabetes(normal)	0.537	1.112	0.795–1.567	–	–	–		
Age-group(55–65)								
66–75	0.001	1.274	1.103–1.476	<0.001	1.711	1.244–2.352		
≥76	0.077	1.342	0.969–1.852	0.043	1.543	1.013–2.349		
Blood pressure(normal)								
sbp>120 or dbp>80	0.173	0.753	0.499–1.132	0.140	1.473	0.886–2.450		
sbp≥140 or dbp≥90	<0.001	2.113	1.581–2.811	<0.001	2.201	1.524–3.178		
Education level(graduate)								
high school diploma	0.111	0.515	0.408–1.380	0.678	0.801	0.282–2.279		
junior diploma	0.334	0.787	0.487–1.271	0.472	0.726	0.306–1.718		
primary school	0.743	1.050	0.797–1.370	0.561	1.240	0.602–2.554		
illiterate	0.075	1.259	0.977–1.623	0.302	1.098	0.506–2.385		
Body mass index(normal)								
thin	0.033	0.666	0.458–0.968	0.033	0.649	0.437–0.966		
overweight	0.343	1.185	0.841–1.642	0.043	1.473	1.013–2.142		
obesity	0.031	1.432	1.033–1.998	<0.001	1.711	1.175–2.490		
Area(mountain)								
township	<0.001	1.580	1.213–2.052	0.700	0.928	1.175–2.490		
urban	<0.001	0.499	0.388–0.643	<0.001	0.456	0.286–0.727		
Diet(middle level)								
nutritionally adequate	<0.001	0.526	0.389–0.711	0.470	0.874	0.286–0.727		
extra serving of meat	0.006	1.532	1.131–2.072	0.861	0.968	0.669–1.401		

For multiple categorical variables, the following were associated with a higher risk of CBVD mortality: those aged 66 to 75 (HR = 1.274, 95% CI = 1.103–1.476); hypertensive patients (HR = 2.113, 95% CI = 1.581–2.811); obesity assessed by BMI (HR = 1.432, 95% CI = 1.033–1.998); and meat-based diet (HR = 1.532, 95% CI = 1.131–2.072). The elderly with a balanced diet were at a lower risk of CBVD mortality than those with an intermediate-type diet (HR = 0.526, 95% CI = 0.389–0.711). Residents who lived in urban areas also had a reduced CBVD mortality rate than the residents who lived in rural (HR = 0.499, 95% CI = 0.388–0.643).

### Results from Multivariate Analysis of the Total Population

In the final model, after all adjustments, the risk of CBVD mortality was associated with increasing age (66–75 age group: HR = 1.711, 95% CI = 1.244–2.352; ≥76 group: HR = 1.543, 95% CI = 1.013–2.349 ). A greater risk of CBVD mortality was due to hypertension (HR = 2.201, 95% CI = 1.524–3.178), being overweight (HR = 1.473, 95% CI = 1.013–2.142) and obesity (HR = 1.711, 95% CI = 1.175–2.490). Female individuals were at a lower risk (HR = 0.6639, 95% CI = 0.457–0.895). The elderly in the normal cognitive function group assessed by MMSE were at a lower risk (HR = 0.650, 95% CI = 0.434–0.973). The risk for urban residents was significantly lower than rural residents (HR = 0.456, 95% CI = 0.286–0.727). Additionally, no significant interactions were demonstrated.

### Results from Multivariate Analysis for Males

In addition, the same analysis was subsequently repeated after further stratification according to gender. The univariate analysis for males showed that depression, disability assessed by IADL, an unhealthy self-assessment, increasing age, hypertension, illiteracy, township, rural residents, and frequent consumption of meat were associated with higher CBVDs mortality. The multivariate analysis showed that self-health assessment, increasing age, and those with hypertension were significantly associated with higher CBVD mortality and inhabitants living in urban areas had a lower risk of CBVD mortality (Table 3).

**Table 3:** Predictors of mortality from CBVDs, using competing risks models for male.

	Univariate analysis			Multivariate analysis				
Variables	*P* value	HR	95% CI	*P* value	HR	95% CI		
Weight	0.214	0.991	0.997–1.010	0.289	1.015	0.987–1.045		
Depression(normal)	0.016	1.673	1.112–2.540	0.150	1.378	0.893–2.127		
Smoke(no smoking)	0.320	0.847	0.611–1.181	–	–	–		
Drink(no drinking)	0.511	0.895	0.641–1.249	–	–	–		
Sad-event(experienced)	0.968	1.011	0.713–1.422	–	–	–		
Excise(experience)	0.341	0.842	0.593–1.202	–	–	–		
BADL(abled)	0.522	1.380	0.543–3.521	–	–	–		
IADL(abled)	0.002	1.810	1.254–2.633	0.478	1.169	0.759–1.802		
Maritalstatus(mateless)	0.960	0.989	0.664–1.473	–	–	–		
MMSE(abnormal)	0.351	0.824	0.549–1.242	–	–	–		
Self-assessment of health(normal)	<0.001	2.211	1.523–.3.24	<0.001	1.894	1.261–2.843		
Blood-lipid(normal)	0.334	0.787	0.488–1.271	–	–	–		
Diabetes(normal)	0.693	0.905	0.553–1.486	–	–	–		
Age-group(55–65)								
66–75	0.012	1.534	1.103–2.125	<0.001	2.233	1.428–3.492		
≥76	0.461	1.152	0.082–1.641	0.023	1.990	1.101–3.596		
Blood pressure(normal)								
sbp>120 or dbp>80	0.284	0.746	0.438–1.271	0.421	1.310	0.682–2.520		
sbp≥140 or dbp≥90	<0.001	2.003	1.410–2.930	0.005	1.924	1.214–3.048		
Education level(graduate)								
high school diploma	0.171	0.496	0.182–1.348	0.759	0.818	0.224–2.981		
junior diploma	0.424	0.808	0.481–1.363	0.608	1.274	0.500–3.247		
primary school	0.683	0.931	0.666–1.312	0.635	1.244	0.497–3.111		
illiterate	0.001	1.730	1.241–2.414	0.654	1.244	0.464–3.340		
Body mass index(normal)								
thin	0.211	0.721	0.432–1.223	0.200	0.702	0.409–1.205		
overweight	0.230	1.300	0.847–2.012	0.008	1.936	1.183–3.166		
obesity	0.471	1.192	0.738–1.922	0.048	1.714	1.004–2.925		
Area(mountain)								
township	0.042	1.438	1.013–2.062	0.210	0.733	0.449–1.197		
urban	<0.001	0.468	0.335–0.654	0.002	0.378	0.202–0.709		
Diet(middle level)								
nutritionally adequate	<0.001	0.452	0.301–0.678	0.330	0.779	0.472–1.287		
extra serving of meat	0.002	1.852	1.257–2.720	0.920	1.025	0.627–1.674		

### Results from Multivariate Analysis for Females

The univariate analysis for females showed that disability assessed by BADL or IADL, without spouse, lower cognitive function, poor self-health assessment, hypertension, obesity, and township or rural living were significantly at a higher risk of CBVD mortality. The multivariate analysis showed that hypertension, a diminished cognition function, and those who had painful emotional and/or mental experiences were associated with increasing CBVD mortality. Living in urban areas was significantly associated with a decreased CBVD mortality (Table 4).

**Table 4:** Predictors of mortality from CBVDs, using competing risks models for female.

	Univariate analysis			Multivariate analysis				
Variables	*P* value	HR	95% CI	*P* value	HR	95% CI		
Weight	0.280	1.011	0.992–1.030	0.441	1.014	0.9781–1.052		
Depression(normal)	0.381	1.213	0.786–1.881	–	–	–		
Smoke(no smoking)	0.442	1.250	0.708–2.213	–	–	–		
Drink(no drinking)	0.763	1.138	0.492–2.633	–	–	–		
Sad-event(experienced)	0.121	0.735	0.497–1.081	0.056	0.674	0.4497–1.010		
Excise(experience)	0.793	1.051	0.719–1.542	–	–	–		
BADL(abled)	0.007	2.480	1.283–4.808	0.250	1.568	0.7238–3.396		
IADL(abled)	0.003	1.781	1.211–2.618	0.719	1.075	0.6380–1.812		
Maritalstatus(mateless)	0.030	1.523	1.043–2.225	0.221	1.331	0.8398–2.108		
MMSE(abnormal)	0.042	0.642	0.417–0.985	0.044	0.602	0.3672–0.985		
Self-assessment of health(normal)	0.017	1.649	1.102–2.502	0.172	1.415	0.8654–2.313		
Blood-lipid(normal)	0.792	1.063	0.705–1.583	–	–	–		
Diabetes(normal)	0.150	1.410	0.883–2.261	0.252	1.325	0.8166–2.150		
Age-group(55–65)								
66–75	0.482	1.152	0.779–1.708	0.286	1.330	0.7724–2.288		
≥76	0.271	1.268	0.830–1.936	0.549	1.264	0.5837–2.737		
Blood pressure(normal)								
sbp>120 or dbp>80	0.374	0.744	0.391–1.410	0.191	1.740	0.7549–4.010		
sbp≥140 or dbp≥90	<0.001	2.365	1.494–3.776	<0.001	2.663	1.4174–5.003		
Education level(graduate)								
high school diploma	0.328	0.496	0.121–2.041	0.196	0.612	0.1031–3.631		
junior diploma	0.169	0.385	0.101–1.482	0.150	0.266	0.0443–1.601		
primary school	0.840	0.948	0.567–1.593	0.551	1.070	0.3200–3.578		
illiterate	0.191	1.347	0.859–2.121	0.132	0.699	0.2122–2.305		
Body mass index(normal)								
thin	0.120	0.646	0.373–1.124	0.126	0.630	0.3447–1.152		
overweight	0.873	1.051	0.617–1.770	0.501	1.187	0.6549–2.151		
obesity	0.014	1.768	1.123–2.869	0.039	1.738	1.0296–2.935		
Area(mountain)								
township	0.003	1.800	1.218–2.662	0.546	1.172	0.5923–2.320		
urban	0.002	0.542	0.369–0.796	0.039	0.489	0.2329–1.028		
Diet(middle level)								
nutritionally adequate	0.041	0.626	0.398–0.981	0.840	0.946	0.5468–1.636		
extra serving of meat	0.452	1.201	0.742–1.950	0.531	0.829	0.4614–1.489		

### Fine and Gray Test

**Figure 2:**
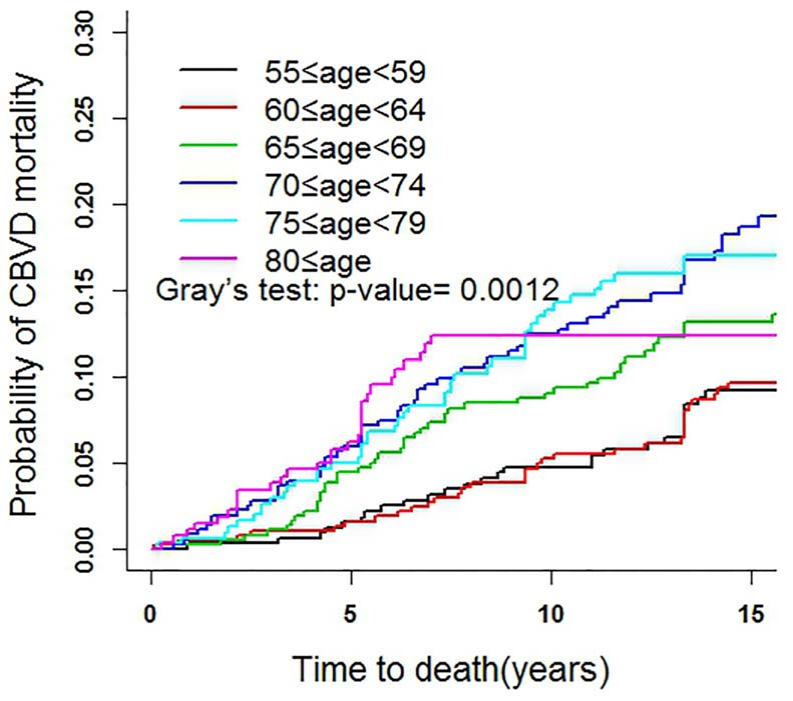
CIFs for CBVDs: comparing different age-groups.

**Figure 3:**
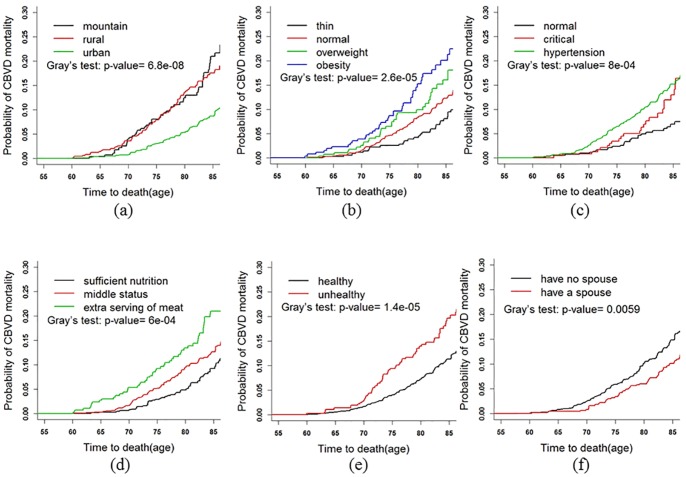
CIFs for CBVDs: comparing different groups after adjusting age.

Gray’s test was used to compare the CIFs of age groups to determine the tendency (Figure 2). The CIF significantly increased with increasing age (*P* value = 0.001). Then Gray’s test was used to analyze other covariates, including gender, marital status, self-assessed health status, disability, depression, cognitive function, *etc*. Age of death was the abscissa to adjust the effect of age distribution in different groups. Several results are shown in Figure 3. As shown in Figure 3a, the CBVD mortality among different residential areas was statistically significant (*P* value<0.001), the elderly who lived in rural areas had the highest CBVD mortality, the CIF at age 85 among rural area arrived at 0.23, township living came second and urban residents were the lowest, less than 0.10. For BMI, obesity was the highest (*P* value = 0.007), and thin scored the lowest (Figure 3b). Those subjects with hypertension or in critical values were at a higher risk of CBVD mortality than normal people (*P* value = 0.001) (Figure 3c). For dietary intake, the mortalities of subjects with balanced diets were the lowest (*P* value<0.001) (Figure 3d). Those with poor self-health assessment were significantly higher than those with healthy self-assessment (*P* value<0.001) (Figure 3e). Individuals who had no spouse scored significantly higher than those who had a spouse (*P* value = 0.006) (Figure 3f). The CIF of smokers was markedly higher than that of non-smokers (P value = 0.057) (not shown). Depression, as assessed by CESD, scored higher than the normal group (*P* value = 0.071) (not shown).

## Discussion

After controlling for competing risks of death from cardiovascular disease, cancer and other causes, results from the present population-based BLSA study showed: gender, cognition function, self-assessment of health, age, blood pressure, BMI, and place of residence, were all found to be independent risk factors associated with CBVD mortality. Univariate analysis and Gray’s test also showed that: diet, marital status, and disability assessed by BADL and IADL were the predictor factors predicting CBVD mortality.

The CBVD mortality was 310.5 *per* 1000,000 among men in China, larger than that among women (242.3 *per* 1000, 000). In our study, the risk of CBVD mortality among females was 0.735 times than that of males. Many epidemiological surveys on the relationship between obesity and CBVDs have shown that obesity, determined on the basis of height and weight by BMI, can become a risk factor when accompanied by hypertension, hyperlipidemia, *etc*., for CBVD [20], [21]. Studies among Chinese adults have also confirmed that elevated BMI increases the risk of both ischemic and hemorrhagic stroke incidence, and stroke mortality, which was similar to our findings [22].

Consistent with previous studies [23]–[25], we also found evidence that closer adherence to the traditional Mediterranean diet, largely based on the consumption of vegetables, fruit, legumes, and olive oil was associated with lower CBVDs incidence and mortality. In our study, those elderly subjects who frequently consumed meat had a significantly higher mortality than the participants consuming more soy-based foods, fruit, vegetables, and fish, as well as participants who more frequently consumed grains (≥0.350 kg *per* person *per* day) and meat (more than twice a week).

Additionally, poorer self-rated physical and mental health status increases the risk of vascular events and mortality in a broad population of patients with symptomatic and asymptomatic atherosclerotic disease [26], therefore indirectly increasing CBVD mortality.

Some studies showed there was no clear evidence that depression is a risk factor for CBVDs [27]. However, a large population-representative cohort confirmed that psychological distress was associated with increased risk of death due to CBVDs [28]. In this study, males who suffered from depression, as assessed by the CESD scale, were at a higher risk than those who did not.

Lots of CBVDs such as cerebral sub cortical small vessel disease, acute stroke, Alzheimer disease, *etc*., were related to cognition in elderly subjects [29]–[31], therefore the lower the cognition according to MMSE, the higher the CBVD mortality. It was confirmed in this BLSA population, that the hazard ratio in the normal group was 0.624 times than the abnormal group assessed by MMSE.

The CIF of CBVD in the rural area was 12.49%, in the suburb it was 13.56%, which was about four to five times than in urban area (5.38%). It may be related with the health systems in urban areas are better equipped than those in the rural areas and townships. Medical resources are sparse in rural areas and the patients cannot get the best treatment: if the patients with a stroke cannot see a doctor in time, they would miss the best treatment opportunities. This is the key public health implication of this research, it suggested that the relevant level of government should rationalize the distribution of medical resources; greater efforts should be made to improve the services provided by community-based medical and health care facilities.

The aging population is increasing worldwide, and CBVD has become the leading cause of death in the elderly. However, few epidemiological studies focused on the aging population. Our study suggested that a balanced diet, frequent exercise, blood pressure control, and a normal BMI, should be encouraged.

Competing risks regression model, which extends Cox’s proportional hazards model to competing-risks data by considering the sub-distribution hazard [32]–[36], does not censor but rather “carries forward” the competing event(s) in the risk set with appropriate weighting [37]–[38]. This study likewise has several limitations. One caveat is that at the end of 2009, 586 subjects were missing, accounting for 29.15% of the population. As this may cause follow up bias. We have compared the differences in characteristics between the missing subjects and the exist subjects, there were no significant differences. Secondly, to avoid the possibility of any clinical or subclinical disease affecting the risk factor status, risk factors in the competing risk model were not updated year by year, only the baseline data were entered into the model.

## Conclusion

This study showed that males were more at risk of having CBVD than females. In addition, increased age, high blood pressure, poor self-evaluated health status, lower cognitive functions, high BMI, a rural primary residence, depression, and more frequent meat intake were associated with higher CBVD mortality of the elderly in Beijing, China.
